# Comparative neonatal outcomes in singleton births from blastocyst transfers or cleavage-stage embryo transfers: a systematic review and meta-analysis

**DOI:** 10.1186/s12958-017-0255-4

**Published:** 2017-05-04

**Authors:** Xingling Wang, Mingze Du, Yichun Guan, Bijun Wang, Junwei Zhang, Zihua Liu

**Affiliations:** The Reproduction Center, The Third Affiliated Hospital of Zhengzhou University, 7 Kangfuqian Road, Zhengzhou, 450052 Henan People’s Republic of China

**Keywords:** Perinatal outcomes, Blastocyst embryo transfer, Cleavage-stage embryo transfer

## Abstract

**Background:**

Comparative neonatal outcomes with respect to singleton births from blastocyst transfers or cleavage-state embryo transfers are controversial with respect to which method is superior. Many studies have yielded contradictory results. We performed a systematic review and meta-analysis for the purpose of comparing neonatal outcomes in single births following IVF/ICSI.

**Methods:**

We searched the Medline, Embase and Cochrane Central Register of Clinical Trials (CCTR) databases until October 2016. Studies and trials that contained neonatal outcomes for singleton births were included. Data were extracted in 2 × 2 tables. The analysis was performed using Rev Man 5.1 software. Risk ratios (RRs) and risk differences, with 95% confidence intervals, were calculated to assess the results of each outcome. Subgroups were applied in all outcomes. Newcastle-Ottawa scale (NOS) checklists were used to assess the quality of the referenced studies.

**Results:**

Twelve studies met the criteria in this meta-analysis. There was a high risk of preterm birth after blastocyst embryo transfer versus the risk after cleavage-stage transfer (RR: 1.11, 95% CI: 1.01–1.22). For the “only fresh” subgroup, the outcome was coincident (RR: 1.16, 95% CI: 1.06–1.27). For the “fresh and frozen” and “only frozen” subgroups, there were no differences. Patients who received fresh blastocyst embryo transfers had a high risk of very preterm births (RR: 1.16, 95% CI: 1.02–1.31). Finally, cleavage-stage embryo transfers were associated with a high risk of infants who were small for gestational age (0.83, 95% CI: 0.76–0.92) and a low risk of those who were large for gestation age (1.14, 95% CI: 1.04–1.25).

**Conclusions:**

The risks of preterm and very preterm births increased after fresh blastocyst transfers versus the risks after fresh cleavage-stage embryo transfers. However, in frozen embryo transfers, there were no differences. Blastocyst embryo transfers resulted in high risks of infants who were large for gestational age, and cleavage-stage embryo transfers resulted in high risks of infants who were small for gestational age.

## Background

Assisted reproduction technology (ART) is frequently employed in modern obstetrics. According to a report by the International Committee for Monitoring Assisted Reproductive Technologies World, the percentage of babies born as a result of ART increased by an estimated average of 9.1% per year from 2008 to 2010. In addition, the global rate of single embryo transfers (SETs) increased from 25.7% in 2008 to 30.0% in 2010 [1].

With the increasing proportion of SETs, neonatal outcomes following SETs are receiving increasing attention, especially with respect to blastocyst transfers versus cleavage-stage embryo transfers. A recent meta-analysis found that cleavage-stage embryo transfers were associated with lower relative risks of preterm births and very preterm deliveries versus risks after blastocyst transfers[2]. Another meta-analysis showed that increased risks of preterm births and congenital anomalies were associated with blastocyst transfers [3]. The most recent meta-analysis indicated that blastocyst transfers were associated with high risks of preterm births, very preterm births and infants who are large for gestational age [4]. However, the reported outcomes of blastocyst transfers are not always consistent. In a study by the reproductive unit of McGill University Health Center, no significant differences in the risks associated with obstetric and perinatal outcomes were found [5]. Another report showed that there was no significant difference in preterm births [6]. Due to these conflicting data, there is an urgent need to provide an update on the available evidence for neonatal outcomes in singleton births after blastocyst transfers versus outcomes after cleavage-stage embryo transfers.

In a recent meta-analysis, fresh embryo transfers were associated with a high risk of preterm births and low birth weights versus risks after frozen embryo transfers [7]. Another meta-analysis also showed that preterm births, low birth weights and rates of infants who were small for gestational age and perinatal mortality were lower in women who received frozen embryos compared to outcomes in those who received fresh embryos [2]. In some studies, however, the blastocyst and cleavage-stage transfer groups combined women who received fresh and frozen embryo transfers, which likely confounded the results. To eliminate this confounding effect, we separated these subgroups.

The objective of this systematic review and meta-analysis was to compare neonatal outcomes in singleton births after blastocyst transfers versus outcomes after cleavage-stage embryo transfer.

## Methods

### Data sources and searches

A systematic literature search was performed on Medline, Embase and Cochrane Central Register of Clinical Trials (CCTR) through October 2016. We searched the literature using the following key words: blastocyst, cleavage, embryo transfer, outcomes, very preterm birth, preterm birth, small for gestational age, large for gestational age, low birth weight and very low birth weight. Two authors (WXL and DMZ) independently conducted the searches and selected the studies to be included. Differences of opinion were resolved after team discussions. Duplicate studies were carefully considered to include comprehensive and high quality studies. Study authors were contacted when more information was needed. Data were extracted using pre-design forms.

### Inclusion criteria

Original studies reporting neonatal outcomes following IVF/ICSI for singleton embryo transfers were included. Studies were sorted by the stage of the embryo at transfer. Cleavage-stage embryos were defined as those at day 2/3/4, and blastocyst embryos were defined as those at day 5/6.

### Exclusion criteria

Studies were excluded if there was no control group, if the neonatal outcomes we needed were not measured, or if there was no independent data from singleton births. Additionally, data gathered after gamete intra-fallopian transfer (GIFT) and preimplantation genetic diagnosis (PGD) were excluded.

### Outcome measures

The included outcomes were infants who were small for gestational age or large for gestational age, preterm births (delivery < 37 weeks), very preterm births (delivery < 32 weeks), low birth weights (birth weight < 2500 g), and very low birth weights (birth weight < 1500 g).

### Statistical analysis

For each outcome, data was extracted in 2 × 2 tables. A meta-analysis was attempted where appropriate. Analyses was performed using the Rev Man 5.1 software (The Nordic Cochrane Center). For binary (or dichotomous) studies, risk ratios (RRs) and 95% confidence intervals were calculated to assess the results of each outcome. A quality assessment of each included study was performed independently by two authors (WXL and DMZ). Any disagreements regarding the type and quality of the studies were resolved via team discussions. The checklists from the Newcastle-Ottawa scale (NOS) were used to assess the quality of studies. If the study had an NOS score ≥ 6, it was regarded as a high-quality study.

### Assessment of heterogeneity

Meta-analyses were performed using random effect models, and we assessed the studies with χ^2^ tests. If an outcome had a low P value (or a large χ^2^ statistic relative to its degree of freedom), this suggested that the evidence had heterogeneity [8]. Additionally, heterogeneity was assessed based on the I^2^ statistic [9]. If the I^2^ value was > 50%, the evidence was deemed as having moderate heterogeneity. A sensitivity analysis was performed by excluding low quality studies (NOS score < 6).

### Assessment of reporting biases

Funnel plots were constructed when an outcome was reported in more than eight studies to test for reporting bias.

## Results

### Results of the searches

**Fig. 1:**
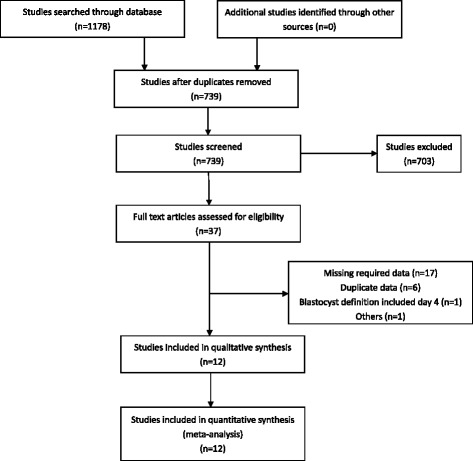
Flowchart for the selection of eligible studies

After searching on Medline, Embase and Cochrane Central Register of Clinical Trials (CCTR) through October 2016, 1178 studies were found (Fig. 1). After reading the titles and abstracts, 37 studies were included. After reading the full text, 12 studies were included in the final analysis. Seventeen studies were excluded because they did not report on the parameters in which we were interested. Three studies [10–12] overlapped with a study from Sweden [13]. In addition, one study [14] and its matched cohort [5] had some duplicate data [15]. One study [16] partially overlapped with another [6]. One study was excluded because it defined day 2/3 as the cleavage stage and day 4/5 as the blastocyst stage [17], which was not consistent with the stage definitions in the other studies. Lastly, one study did not separate the blastocyst and cleavage stages in some aspects [18], and the data were not in the format we needed.

### Included studies

Twelve studies were included in this analysis. They compared neonatal outcomes in singleton births after blastocyst transfers (day 5/6) versus outcomes after cleavage-stage embryo transfers (day 2/3/4) after IVF/ICSI.

### Methodology of the included studies

Twelve retrospective unmatched cohort studies were included. All studies scored high (≥6) on NOS checklists. Data were pooled from databases except in one study [19].

### Populations in the included studies

Despite strict inclusion criteria, there was still variation in the populations of the 12 studies. Most of the studies included patients undergoing IVF/ICSI, but two studies included couples only undergoing IVF [20, 21]. Four studies [6, 13, 22, 23] confounded fresh and frozen embryo transfers, while two clearly separated those variables [13, 23]. Others included only fresh embryo transfers.

### Exposure in the included studies

In the 12 studies, cleavage-stage transfer was defined as transferring the embryo on day 2/3/4, and blastocyst transfer was defined as transfers on day 5/6. These details are included in Table 1.

**Table 1:** Characteristic of included studies

Study	Year	Source	Patientor population	Method of data collection	Risk of bias	Outcomes	NOS scoring
Chambers, et al. (2015) [6]	2009 to 2012	National registry	47370 live deliveries in Australia and New Zealand (day 2/3 vs day 5/6)	Australian and New Zealand Assisted Reproduction Database	Some patients do not have complete information	No increased risk of LBW and PTB resulting from blastocyst transfers compared to cleavage transfers	8
Dar, et al. (2013) [24]	2001 to 2009	National registry	12712 singletons in Canada on a voluntary basis (day 3 vs day 5/6)	Canadian ART Register database	The PTB unadjusted by potential confoundding factors. The women in the blastocyst group were young	Increased risk of preterm birth with day 5/6 transfers	8
Fernando, et al. (2012) [22]	2004 to 2009	Single center	4202 women conceived via IVF/ICSI in Australia (day 2/3/4 vs day 5/6)	Monash IVF patient database	Cleavage stage includes day 4.	No statistically significant difference between transfers on days 5/6 and days 2/3/4 in all maternal and perinatal outcomes	7
Ginstrom Ernstad, et al. (2016) [13]	2002 to 2013	National registry	30566 singletons in Sweden via IVF/ICSI treatments (day 2/3 vs day 5/6)	Swedish Medical Birth Register and the National Patient Register	The number of blastocyst transfers is very low relative to the number of cleavage-stage transfers	Singletons born after blastocyst transfer had a lower risk of LBW and SGA as compared to cleavage-stage transfers.	8
Kalra, et al. (2012) [20]	2004 to 2006	National registry	69039 live deliveries via IVF in U.S. (day 3 vs day5/6)	Society of Assisted Reproductive Technologies database	Women in the blastocyst transfer group were young. No ICSI cycles in the study.	After blastocyst transfers, patients were at an increased risk for PTB and VTPB as compared with cleavage-stage transfer	9
Martin, et al. (2012) [19]	2002 to 2009	Single center	1183 singltons from the hospital of Tours, France (day 2 vs day 5/6)	Forms completed by couples	Forms filled out by couples.	Incresded risk of PTB after blastocyst transfer	7
Oron, et al. (2014) [15].	December 2008 to December 2012	Single center	1543 single embryo transfers in McGill University Health Center, Canada (day 2/3 vs day 5)	In their computerized database	The small number of live births resulting from cleavage and blastocyst embryo transfers.	No increased risk of maternal or neonatal complications in pregnancies resulting from blastocyst embryo transfers	8
Maxwell, et al. (2015) [25]	2003 to 2012	Single center	392 singleton live births via IVF/ICSI at New York University Fertility Center (day 3 vs day 5/6)	In their computerized database	Women were young in the blastocyst group.	No increased risk of PTB and VPTB	8
Zhu, et al. (2014) [21]	January 2009 to June 2012	Single center	2929 singletons born at Peking University Third Hospital (day 3 vs day 5/6)	In their computerized database	The number of day 3 transfers was high relative to the number of day 5/6 transfers. No ICSI cycles in the study	No increased risk of SGA	8
Ishihara, et al. (2014) [23]	2008 to 2010	National registry	277042 singletons born in Japan	Japanese ART registry database collected by the Japan Society of Obstetrics and Gynecology	Women were young in the blastocyst transfer group.	Blastocyst transfers were associated with a significantly decreased rate of SGA	8
De Vos, et al. (2015) [27]	April 2004 to December 2009	Single center	2098 singleton live births in single center, Belgium (day 3 vs day 5)	In their computerized database	Women were young in the blastocyst group.	The mean singleton birthweights were not different between day 3 embryo transfers and day 5 blastocyst transfers	8
Makinen, et al. (2012)	2000 to 2010	Single center	1079 infants born after treatment at the Family Federation of Finland Fertility Clinic in Helsinki, Finland (day 2/3 vs day 5/6)	In their computerized database	Details about patients were not clearly described.	No increased risk of SGA	7

## Results of the outcome measures

### Preterm birth (delivery at < 37 weeks)

**Fig. 2:**
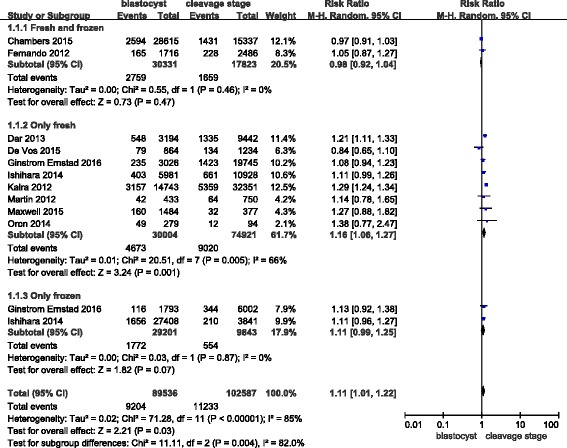
Meta-analysis of blastocyst embryo transfers versus cleavage-stage embryo transfers for preterm births (<37 weeks)

Ten studies covered preterm births (delivery at < 37 weeks). The RR of delivery at < 37 weeks was 1.11 (1.01–1.22) in singleton births after blastocyst transfers compared with the risks after cleavage-stage transfers (Fig. 2). The Q statistic P-value was < 0.00001, and the I^2^ statistic = 85%, indicating high heterogeneity. For all of the studies with a high NOS score, a sensitivity analysis did not improve the outcomes.

As mentioned in the introduction, fresh embryo transfer is associated with a high risk of preterm birth. We attempted to parse out the confounding factors to further explore this assessment, but unfortunately, two studies mixed fresh and frozen embryos in their analyses. Therefore, we do not know the exact proportions of fresh/frozen embryos in the blastocyst/cleavage-stage groups for these studies; hence, we set up three subgroups for our analysis: “fresh and frozen”, “only fresh” and “only frozen”. In the “only fresh” subgroup, the RR of delivery at < 37 weeks was 1.16 (1.06–1.27). In the “fresh and frozen” subgroup, the RR of delivery at < 37 weeks was 0.98 (0.92–1.04). Finally, in the “only frozen” subgroup, the RR of delivery at < 37 weeks was 1.11 (0.99–1.25). Based on these data, we concluded that there was no significant difference between the “only frozen” and “fresh and frozen” subgroups. However, in the “only fresh” subgroup, a high risk of preterm birth after blastocyst embryo transfer versus the risk after cleavage-stage embryo transfer was observed.

**Fig. 3:**
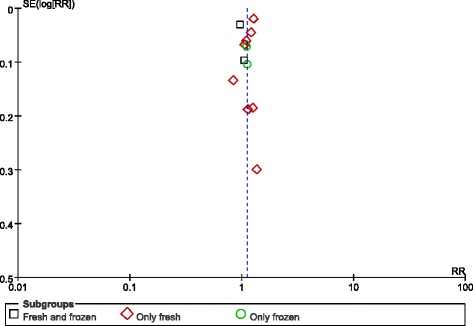
Funnel plot of studies reporting preterm births

Lastly, no publication bias was found in the funnel plot (Fig. 3).

### Very preterm birth (delivery at < 32 weeks)

**Fig. 4:**
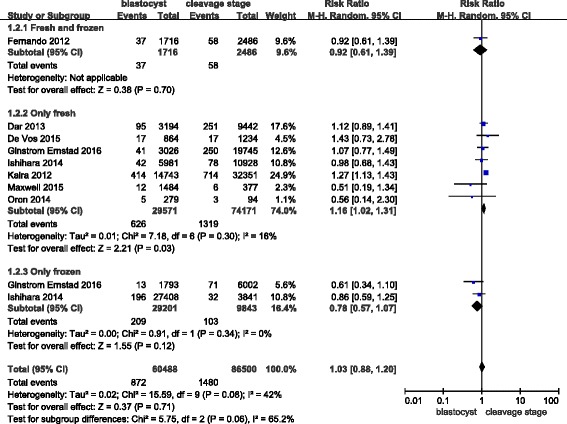
Meta-analysis of blastocyst embryo transfers versus cleavage-stage embryo transfers in relation to very preterm births (<32 weeks)

Eight studies covered very preterm births (delivery at < 32 weeks). The RR of delivery at < 32 weeks was 1.03 (0.88–1.20). The results of the outcome analysis indicate that the risk of delivery at < 32 weeks was similar in singleton births after blastocyst transfers and cleavage-stage transfers. However, we found that the risk of a very preterm birth became higher after a fresh blastocyst transfer versus after a fresh cleavage-stage transfer (RR: 1.16, 95% CI: 1.02–1.31), but no differences were found between the “fresh and frozen” and “only frozen” subgroups (Fig. 4).

**Fig. 5:**
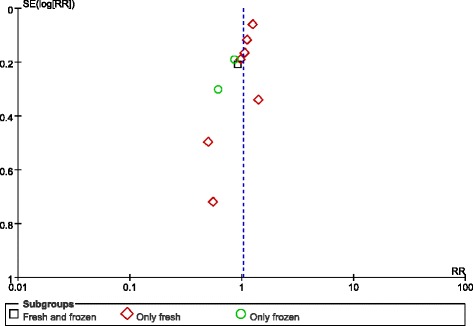
Funnel plot of studies reporting very preterm births

The funnel plot did not demonstrate any publication bias (Fig. 5).

### Low birth weight (birthweight < 2500 g)

**Fig. 6:**
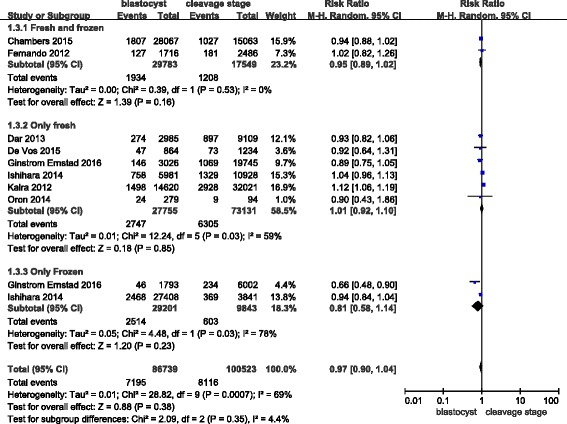
Meta-analysis of blastocyst embryo transfers versus cleavage-stage embryo transfers in relation to low birth weights (<2500 g)

Eight studies covered low birth weights, defined as < 2500 g. The RR of a birthweight < 2500 g was 0.97 (0.90, 1.04) in singleton births after blastocyst transfers compared with the risk after cleavage-stage transfers (Fig. 6). The Q statistic P-value = 0.0007, and the I^2^ statistic = 69%, indicating high heterogeneity. For all of studies with a high NOS score, a sensitivity analysis did not improve the outcomes.

As mentioned in the introduction, fresh embryo transfer was associated with a high risk of a low birth weight. Therefore, we used the same method as in analyzing preterm births. In the “only fresh” subgroup, we found that the RR of a low birth weight was 1.01 (0.92–1.10). In the “only frozen” subgroup, the RR of a low birth weight was 0.81 (0.58–1.14). In addition, the RR of the “fresh and frozen” subgroup was 0.95 (0.89–1.02).

**Fig. 7:**
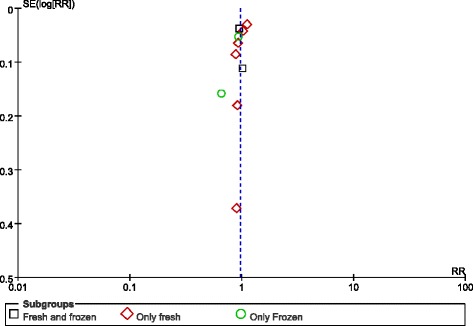
Funnel plot of studies reporting low birth weights

The funnel plot did not show any publication bias (Fig. 7).

### Very low birth weight (birthweight < 1500 g)

**Fig. 8:**
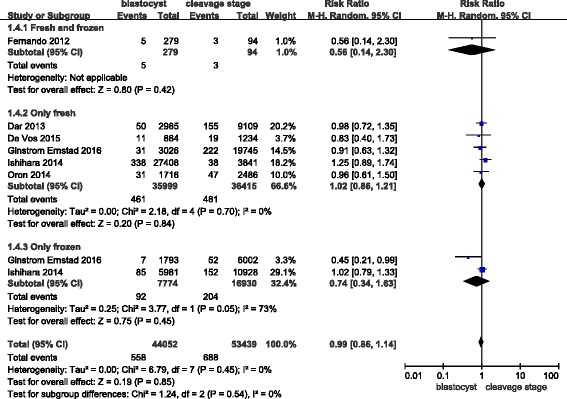
Meta-analysis of blastocyst embryo transfers versus cleavage-stage embryo transfers in relation to very low birth weights (<1500 g)

In our meta-analysis, six studies covered very low birth weights. The RR of a very low birth weight was 0.99 (0.86–1.14) in singleton births after a blastocyst transfer compared with the risks after a cleavage-stage transfer. In addition, the Q statistic P-value = 0.45, and the I^2^ statistic = 0%. The results indicate that there was no increased risk of a very low birth weight after blastocyst transfer versus the risk after a cleavage-stage transfer (Fig. 8).

### Small for gestational age (< 10th percentile or < -2 SD)

**Fig. 9:**
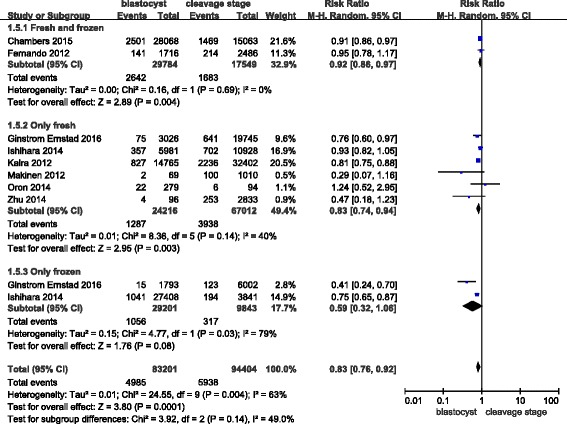
Meta-analysis of blastocyst embryo transfers versus cleavage-stage embryo transfers in relation to infants who were small for gestational age

In this meta-analysis, eight studies covered small for gestational age outcomes. However, the definition of this outcome was different across studies. Most studies defined it as < 10th percentile on the intrauterine growth chart. However, small for gestational age was defined as < -2SD in this study. The RR of infants being small for gestational age was 0.83 (0.76–0.92) in singleton births after a blastocyst transfer when compared with the risk after a cleavage-stage transfer (Fig. 9). The Q statistic P-value = 0.04, and the I^2^ statistic = 63%, which indicates high heterogeneity. For all studies with a high NOS score, a sensitivity analysis did not improve the results.

As discussed previously, fresh embryo transfers are associated with a high risk of infants being small for gestational age; so, we employed the same analytical methods as for preterm births. In the “only fresh” subgroup, we found that the RR of infants being small for gestational age was 0.83 (0.74–0.94), and there was low heterogeneity. In the “only frozen” subgroup, the RR of infants being small for gestational age was 0.59 (0.32–1.06). The RR for the “fresh and frozen” subgroup was 0.92 (0.86–0.97).

**Fig. 10:**
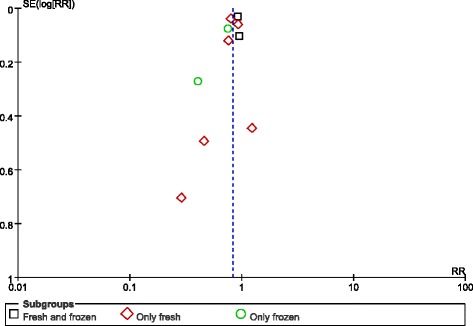
Funnel plot of studies reporting infants who were small for gestational age

The funnel plot did not reveal any publication bias (Fig. 10).

### Large for gestational age (> 10th percentile or > -2 SD)

**Fig. 11:**
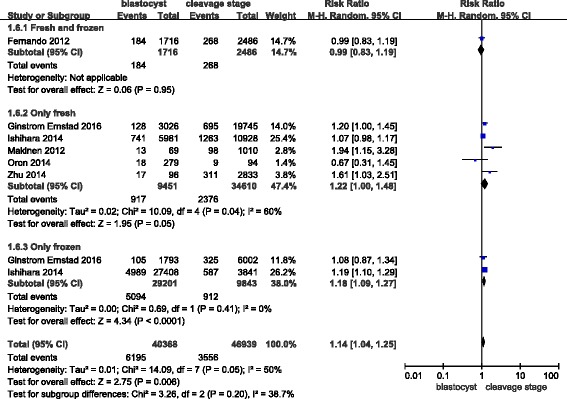
Meta-analysis of blastocyst embryo transfers versus cleavage-stage embryo transfers in relation to infants who were large for gestational age

In this meta-analysis, six studies covered large for gestational age outcomes. The RR of infants being large for gestational age was 1.14 (1.04–1.25) in singleton births after a blastocyst transfer compared with the risk after a cleavage-stage transfer (Fig. 11), and the Q statistic P-value = 0.05, with a corresponding I^2^ statistic = 50%. These results indicate that there was no increased risk of a large for gestational age outcome after a blastocyst transfer compared with the risk after a cleavage-stage transfer.

## Discussion

### Main findings

In our meta-analysis, we observed a higher risk for both preterm births and very preterm births after blastocyst embryo transfers versus the risks after cleavage-stage embryo transfers. We did not find obvious differences in frozen embryo transfer outcomes. In addition, we found a decreased risk of infants being small for gestational age associated with blastocyst embryo transfers and an increased risk of infants being large for gestation age associated with cleavage-stage embryo transfers. Finally, we did not find an association between blastocyst embryo transfers and cleavage-stage embryo transfers in relation to the other aspects of our study.

### Strengths

Up to now, two systematic meta-analyses have analyzed the outcomes of blastocyst and cleavage-stage embryo transfers. One compared obstetric and perinatal outcomes between the two groups, and the other compared only neonatal outcomes. We included twelve studies in our systematic meta-analysis, creating a larger data set than either of the two previous studies performed.

In addition, we analyzed whether embryos used either frozen or fresh affects these outcomes. Aside from the two meta-analyses we mentioned previously, we also found a recent study concluding that birth weight (frozen versus fresh: 3310.6 ± 579.5 versus 3243.7 ± 558.6, *P* = 0.008) and the frequency of infant being small for gestational age (frozen versus fresh: 6.6% versus 10.2%, *P* = 0.005) had different outcomes in singleton births [18]. In summary, we concluded that mixing fresh and frozen embryos into the same comparison group is a confounding factor.

### Limitations

Although our study inclusion criteria were strict, there was still variability between the studies we used, indicating that our meta-analysis possesses some limitations.

In several studies, the days compared for embryo transfer were different. For example, in one study, days 2–4 was compared with days 5–6 [22]. Two studies compared day 3 with days 5–6 [20, 21, 24, 25]. Additional studies compared days 2–3 with days 5–6 [6, 13, 26], and one study failed to mention the exact days that were compared altogether [23]. The last three studies compared day 2 with days 5–6 [19], days 2–3 with day 5 [15], and day 3 with day 5 [27]. An additional confounder in some studies arose as a result of including frozen embryo transfers and fresh embryo transfers in the same comparison group [6, 13, 22, 23]. It is also difficult to know the quality of the embryos and the method that was employed for freezing embryos in each study. The outcomes of vitrified embryo transfer and slow-freezing embryo transfer are different. One study showed that the median birth weight of babies born from slow-freezing embryos is lower than that of those born from vitrified embryos [28]. In a previous study, they included patients in their center from January 2006 to May 2011. For the singleton birth group, birth weight (3455.3 ± 482.0) after vitrified embryo transfer was higher than after slow-freezing embryo transfer (3352.3 ± 500.7) (*P* = 0.0001), and the media for extending embryo cultures was also different.

In addition, the patients in these studies had variant characteristics, such as maternal age, parity, smoking, body mass index, years of involuntary childlessness, and history of preterm birth. For example, the mean age of patients at the time of blastocyst transfer was younger than for those undergoing cleavage-stage transfer in some studies [6, 15, 20, 22, 24]. In one study, however, there was no difference in the mean age between these groups [19]. Without individual data, we cannot eliminate these potentially confounding variables. Definitions for outcomes also differed in some instances between different studies, and some studies may have used donor oocytes. For some subgroups, the number of included studies was small. We need more data to eliminate the risk of bias.

Finally, we used retrospective cohort studies. Data from randomized, controlled trials would be more reliable in assessing these outcomes.

### Clinical implications of the study

In our meta-analysis, we found a high risk of preterm birth after blastocyst embryo transfer versus the risk after cleavage-stage embryo transfer, especially in the “only fresh” subgroup. In the other subgroups, however, there were no differences. We also observed that fresh blastocyst embryo transfer had a high risk of very preterm birth. In addition, people who underwent blastocyst embryo transfers were at a low risk of having infants who were small for gestational age and a high risk of having infants who were large for gestation age versus the risks following cleavage-stage embryo transfers. We do not have an absolute conclusion as to which method of embryo transfer is superior for patients; so, we decided to expand our study to also include pregnancy outcomes. A recent study found that live birth rates per started cycle (31.3% versus 37.8%, *P* = 0.041) were significantly lower after transferring fresh single cleavage-stage embryos compared to rates after transferring blastocysts.

However, the cumulative live birth rates (52.6% versus 52.5%, *P* = 0.989) were not significantly different between cleavage-stage and blastocyst embryo transfers [29]. Additional studies have reached the same conclusion as professor De Vos. In a recent meta-analysis that included five randomized control trials, it was found that there was no difference in cumulative pregnancy rates (0.82, 0.60–1.12) between cleavage-stage and blastocyst embryo transfers [30].

In most cases, cleavage-stage transfers result in more available embryos, and blastocyst transfers may confer a lesser chance of a successful transfer. Whether either method is better than the other remains to be determined.

## Conclusion

For many of the aspects we investigated, randomized, controlled trials are needed to more accurately assess the outcomes. In addition, studies where embryos are of an unknown status in terms of whether they are frozen or fresh present the potential for confounding effects. Of course, the reasons for differential outcomes could also improve the efficacy of ART. In addition, follow-up investigations need to be updated to include babies’ growth and developmental progress.

## Abbreviations

ART: Assisted reproduction technology
ART: Assisted reproductive technology
ICSI: Intracytoplasmic sperm injection
IVF: In vitro fertilization
LBW: Low birth weight
PTB: Preterm birth weight
SET: Single embryo transfer.
SGA: Small for gestational age
VPTB: Very preterm birth weight
